# A Telehealth System Incorporating a Serious Game Intervention to Aid Occupational Therapists in Identifying and Treating Children With Difficulty Crossing the Body’s Midline: Key Informant Interviews Among Occupational Therapists

**DOI:** 10.2196/27761

**Published:** 2021-11-01

**Authors:** Jonathan Jacobs, Reolyn Heymann, Jacob Jacobus Greeff

**Affiliations:** 1 Centre for Collaborative Digital Networks Department of Electrical and Electronic Engineering Science University of Johannesburg Johannesburg South Africa; 2 School of Computer Science and Information Systems Faculty of Natural and Agricultural Sciences University of North-West North-West South Africa

**Keywords:** serious games, input device, telehealth, occupational therapy, midline crossing

## Abstract

**Background:**

The midline is an imaginary line that isolates the left and right parts of the body. Crossing the midline infers that a body part (eg, hand or foot) can spontaneously move over to the opposite side of the body to perform an action. A child who has difficulty crossing the midline can physically perform actions that cross the center of the body; however, they do not intuitively cross the midline when challenged with a task that requires this movement, as their perceptual components prevent them from engaging on the contralateral side. This requires treatment from an occupational therapist. Owing to the recent COVID-19 pandemic, access to therapeutic sessions was not possible or reduced, putting the responsibility for treatment on caretakers at home. Caretakers do not have the knowledge and skills to provide treatment, and occupational therapists do not receive adequate feedback from caretakers on the child’s progress.

**Objective:**

The first objective is to adapt a simple serious game, or applied game, into a telehealth solution. Children will play the game at home under the supervision of a caretaker, and the results will be stored on the web. Occupational therapists can monitor progress via a web-based dashboard, receive additional valuable feedback about the child’s behavior during treatment, and easily adapt the game to target specific needs. The second objective is to evaluate whether the implemented telehealth solution is feasible as a treatment option for midline crossing difficulties and thus fit for purpose.

**Methods:**

To meet the first objective, engineering and game development stakeholders formed a team with an occupational therapist, and through a collaborative design process combined with an agile programming approach, a telehealth solution was designed to assist remote monitoring of the serious gameplay. For the second objective, 6 different occupational therapists were introduced to the game, had the opportunity to play the game, and then provided feedback regarding the feasibility, benefits, and applicability of the system during structured interviews.

**Results:**

A telehealth system was designed aimed to address this problem. All results are saved on the web and accessed by occupational therapists via a dashboard. In addition, observed behavioral information is also saved. During the interviews, occupational therapists indicated that the dashboard would support their treatment plan and was indeed a feasible solution.

**Conclusions:**

The feedback from the occupational therapists for this telehealth solution suggests a feasible method to treat midline crossing problems remotely. The therapists commented on the convenience of integrating both assessment and treatment into the same application, as it assists them when grading a child. The therapists collectively agreed that the quantitative aspect the serious game creates by providing measurable and standardized data proves advantageous when compared with traditional methods of assessment and treatment.

## Introduction

### Background

Owing to the COVID-19 pandemic, numerous health care practitioners were unexpectedly required to transition their standard in-person treatment to telehealth options, often without advance preparation or training [1]. Telehealth is becoming an increasingly used service delivery model in rehabilitation services. Telehealth has the potential to alleviate provider shortages, decrease costs associated with providing therapy, and allow for treatment within a client's natural environment [2]. Telehealth can be used by occupational therapists for evaluation, intervention, education, and prevention of injury or exacerbation of conditions [3]. Telehealth facilitates collaboration and consultation with other professionals, which facilitates coordination of care [4].

The midline is an imaginary line that isolates the left and right parts of the body. Crossing the midline infers that a body part—for example, a hand or foot—can spontaneously move over to the opposite side of the body to perform an action. To clarify, a child who has difficulty crossing the midline can physically perform actions that cross the center of the body; however, they do not intuitively cross the midline when challenged with a task. The diagnosis of children with midline crossing difficulties requires a cluster of clinical observations that indicate bilateral integration dysfunction [4]. A study was performed in which 10% of the sample was considered to constitute a possible deficit range, and a further 10% was considered to be in the suspect range. The remaining 80% was considered to fall in the normal range [4].

Midline crossing is a difficulty that needs the intervention of an occupational therapist. Midline crossing difficulties may affect the child’s physical well-being as well as their future development if it is not addressed in a timely manner. If a child does not get the needed intervention due to the COVID-19 lockdown, it can affect the child’s future. Being able to cross the midline is a developmental milestone. By the age of 5 years, a child is expected to be able to cross the midline, that is, use both sides of the body simultaneously [5]. When the midline is crossed spontaneously, supporting neural networks and pathways for specific activities are developed. This is a prerequisite skill essential for the development and maintenance of motor and cognitive demands associated with specific activities. Consequently, children who have trouble crossing the body’s midline also frequently experience difficulty with reading, writing, tying their shoelaces, brushing their teeth, and participating in physical activities [6].

Current treatment approaches used by therapists are arts and crafts in which actions such as threading beads, cutting, pasting, and folding paper are used as these actions require the midline to be crossed. Finger puppets or stickers are also used by placing or sticking the puppets or stickers on one of the child's hands and then encouraging the child to remove the puppet or sticker with the opposite hand. Other common methods used by therapists include building blocks and playing Twister and marching games using arms and legs. Occupational therapists use standardized assessment tools such as the Movement Assessment Battery for Children, the Developmental Test of Visual Motor Integration, and the Draw a Person Test to measure changes in function and occupational. These standardized tests have procedures for administration and scoring [7]. However, when measuring the outcome of an intervention, there is still a lack of tools available for objective measurement, particularly among children with perceptuomotor or attention deficit disorder [7].

At present, occupational therapists observe if the child exhibits the actions given in Textbox 1.

**How to identify whether a child has difficulties crossing the midline.**

**How to identify whether a child has difficulties crossing the midline**

- Swaps hands midway through a task when writing, drawing, painting, or coloring
- Uses the left hand for activities on the left side of the body and right hand for activities on the right-hand side
- Rotates their trunk to the opposite side when reaching across the body (to avoid crossing the body midline)
- Has difficulty visually tracking an object from one side of the body to the other, such as following text when reading
- Has poor pencil skills (pencil grip)
- Uses different feet to kick a ball (mixed dominance)
- Has difficulty coordinating gross motor patterns (eg, crawling, skipping, and star-jumps)

On the basis of their observations and discretion, they conclude whether the child has difficulty crossing the midline. Assessment is an integral part of the occupational therapy process and is a necessity for evidence-based practice. Without appropriate measurement, therapists cannot provide evidence for the offered interventions [8]. The assessment is subjective, and there are issues with this subjectivity, as the same level of difficulty may be rated differently by different assessors. When therapists write reports for the child, it is challenging to justify the child's improvement as there is no criterion to compare it with. In addition, numerous children up to the age of 5 years living in underdeveloped countries, including South African rural areas, face exposure to multiple risks affecting their early childhood development [8]. These children and their parents are often not aware of the functional difficulties they may have, and even if they are aware, they need to travel long distances to receive the therapy they require. Moreover, because of the recent COVID-19 pandemic and resulting lockdowns, many children who would normally receive occupational therapy could not access occupational therapists or had to decrease their sessions.

If a child has a pathology of midline crossing, frustration in the behavior of the child will be noticeable, as the child will become angry when trying to engage in fine motor activities because of less refined hand skills. The coordination of both sides of the body will be less refined, leading to difficulties experienced when playing sports or doing any physical activity. In addition, when children have difficulty with midline crossing, they may additionally have trouble visually tracking an object from left to right. Thus, when the object reaches the midline, they often blink and have to refocus, and this results in their losing their place while reading. In addition, when drawing horizontal and diagonal lines as well as writing letters such as an *x*, they may segment these lines rather than overlap them because of midline crossing difficulties [9]. Some children may struggle to cross the body’s midline easily. When a child shows hesitancy in reaching, stepping, or looking across the midline of the body, it is known as midline crossing inhibition. Sometimes, this delay can be seen when a child hesitates or is clumsy during gross motor tasks that require the arm or leg to cross over to the other side. Some children with delayed midline crossing skills may display some *compensatory mechanisms* in school that make writing awkward for them. Crossing the midline is a treatable affliction. If a child struggles to cross the body's midline and is treated, milestones such as developing a dominant hand will occur [6]. Pencil skills and fine motor tasks will be refined, easing the transition to an academic environment where those skills are expected to be grasped [6]. The child will be able to complete self-care tasks, for example, brushing their teeth and getting dressed. The child will be able to kick and hit balls as well as run as their gross and fine motor skills will be improved. Finally, their ability to visually track across a page effectively will be better and, therefore, will result in fluent reading.

At the forefront of technological advancements in occupational therapy are serious games [10]. For the purpose of this research, serious games are described, among other things, as digital games, virtual environments, simulations, and a mixed reality that engage the player. These serious games form encounters and experiences that convey meaning [11]. Serious games are introduced as a need to meet objectives that go beyond entertainment and benefit the user in the area that needs to be mitigated. The applications created under the terminology of serious games induce motivation and engage the user [12].

### Objective

Before proposing how serious games can be integrated, a major obstacle to treating children in the conventional sense is boredom because of the intensive and repetitive practice required. The advantage of incorporating input devices and the use of serious games into treatment methods is that it combats the boredom factor. The child is placed into a game environment that is similar to the real world in terms of the perceptual stimuli it exhibits, which then puts the child at ease [13]. The child then has the ability to manipulate and control some of the stimuli and see the outcome of their actions in real time and adjust them accordingly. This aspect, being the interactive component, creates an engagement with the environment, allowing the child to feel and be in control of their movement. This idea is explained as *the perceptual illusion of nonmeditation* [14]. The sensations that are familiar and present when playing the game and the ability of the child to control and manipulate the stimuli that surround them generate the psychological effects of enjoyment and, particularly, involvement [14]. The unique merging of purpose and pleasure develops intrinsic motivation in the child.

Microsoft’s Kinect sensor has been highly investigated and used for the development of new complements that help improve or optimize rehabilitation processes worldwide. In 2012, Ruiz and Cantos [15] designed a therapeutic tool using the Kinect for neurorehabilitation using games to stimulate patients, cognitive functions, perceptions, and gross and visual motor skills through play. This tool verified that patients had fun while being treated in this manner; intrinsic motivation is achieved by merging purpose and pleasure.

Moreover, using the Kinect and combining purpose and pleasure, Chang et al [16] performed a study in Taiwan in 2013 that proposed the possibility of rehabilitating two 14-year-old adolescents with cerebral palsy through therapies personalized to their condition. Data showed that the 2 participants had significantly increased motivation for upper limb rehabilitation, thus improving exercise performance during the intervention phases.

Although Kinect is a useful device, it can, however, encounter the problem of misdetection when it comes to extremity angles or overlapping extremities [17]. In 2016, the Taiwan University proposed a new rehabilitation gaming system, which focuses on the upper part of the body with wireless inertial measurement units (IMUs) and a Kinect device. The Kinect was used as a base tracking system by the gaming system. Multiple sets of IMUs were integrated into the extremity of the subject to calculate the angles through algorithms. Wireless IMUs were also added to compensate for the error in the calculation of angles in the Kinect device [17]. This study exposed that the use of the Kinect by itself may not be sufficient and, therefore, additional sensors were needed to ensure correct motion capture.

Nintendo’s Wii Fit is a commercial product used for both fitness and fun and was created to encourage people to exercise as well as improve balance. The Wii Fit uses the Nintendo Wii console and a balance board. A study conducted by the University of Naples found that the Wii Fit showed better improvements regarding physical therapy in terms of balance and self-confidence than conventional treatment [18]. It was concluded that the Wii Fit is acceptable as an adjunct to virtual rehabilitation interventions and provides an exciting new therapy device [18]. The Wii console does not provide a platform or offer a game in which more specific impositions, such as difficulty crossing the midline, can be treated. If one wanted to use the Wii for treatment or training purposes, one would need to purchase not only the balance board but also the console and remote, thus making it too expensive for the communities that this study is aimed at.

Ultraleap’s Leap Motion Controller is a computer hardware sensor device analogous to a mouse [19]. Using motion capture technology, Leap Motion is able to process the input. It does not require direct hand contact with the device as input; instead, hand and finger motions are tracked. Despite the small size of Leap Motion, it is capable of capturing smaller details, such as finger movement. Leap Motion is smaller and cheaper than Kinect; however, Kinect is more precise in capturing movement. Sourial and Reichardt [20] proposed implementing a virtual therapist (VT) to help patients do their exercises at home in an engaging gamified environment. The VT artificial intelligence used a hierarchical finite-state machine architecture. Hand therapy helps the patient regain the hand's full functionality after a certain injury or surgery. Hand therapy could be a very tedious process that implies physical exhaustion [20]. In addition, finding appointments with the therapist frequently enough for an efficient healing process is difficult and costly. To test the efficiency of the VT, a web-based hand therapy exercise was implemented using the Unity platform to build the exercise environment. Leap Motion technology was used to detect the information of the hand movement. This exercise was tested on 19 participants. The idea of being coached by a VT was welcomed by the participants, as the exercise was fun and motivating to them. VT guidance and assessment were helpful and easy to follow. However, some modifications are needed in the pain detection part to form a more efficient exercise [20].

Another system using Leap Motion and capable of improving fine motor skills in children, was proposed by Hidalgo et al [21] through a serious game and 3D environment. The proposed system allowed the therapist to choose among different levels of serious games according to the child’s needs. The game excited the children; however, the children took time to adapt to the game because of the inaccurate readings of Leap Motion. Therefore, it can be noted that including different levels creates excitement for the children as they experience different challenges; however, when technology interferes with the game or is difficult to use, the child takes time to become familiar with the system.

Sony’s motion capture system Intel RealSense enables fine-motoric gesture recognition, and its small form factor allows for preintegration into notebooks and tablets, substituting conventional cameras [22]. This setup enables new methods of therapy in the form of serious games that are engaging and easy to set up. Chhor et al [22] developed and evaluated a serious game prototype for rehabilitation using Intel's RealSense (called *Breakout*) based on a commercial game framework. Despite the fact that RealSense can easily integrate with the applications mentioned above, it is an expensive app.

Neuroplasticity refers to the ability of the brain to adapt structurally and functionally and is enhanced by training and experience. It is known that neuroplasticity is at its maximum in a critical period, which corresponds to the first 7 years of a child’s life [23]. Therefore, with the development of a serious game and given the neuroplasticity of the child’s brain at this age, the child may be able to develop the required pathways necessary to conduct movements that once seemed impossible. With the integration of technology and the innovation and creativity of this approach, a beneficial method of mitigating impositions such as midline crossing could be developed to assist occupational therapists in the treatment of children. A serious game with an input device was initially developed to assist in midline crossing therapy [24]. An improved wireless input device that accompanied the serious game was developed [25]. The aim of this study is twofold. The first objective focuses on the design of a web-based telehealth system that consists of a serious game, an input device, and a web-based dashboard that displays relevant data to occupational therapists. The second objective focuses on whether the system is fit for its purpose and can be adopted by occupational therapists to treat patients remotely.

## Methods

### Objective One: Designing a Remote Monitoring System for a Midline Crossing Serious Game

#### Overview

**Figure 1:**
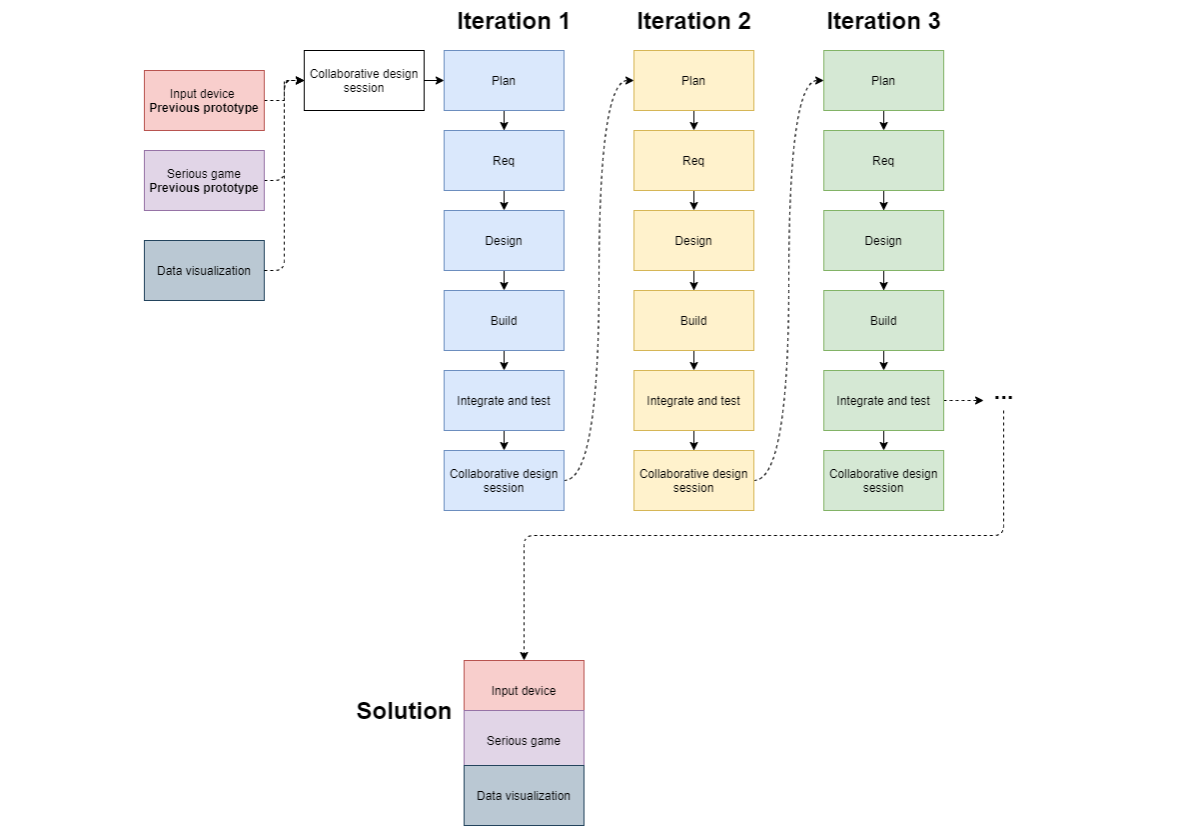
Iterations in the agile software development life cycle. Req: requirements.

An agile software development life cycle approach was taken to develop the end-to-end solution. This methodology was chosen because of the frequent feedback needed during the design process, allowing the design team to give recommendations through collaborative design at the end of each iteration. The design team comprised game designers, electronic engineers, and an occupational therapist. Figure 1 shows the iterations of the agile software development life cycle. It should be mentioned that each component of the solution (serious game, input device, and telehealth system) was developed separately through iterations; however, the components were integrated and tested at the end of each iteration. Furthermore, the initial serious game from the previous project was used as a foundation to produce the web-based telehealth solution presented in this study [24].

**Figure 2:**
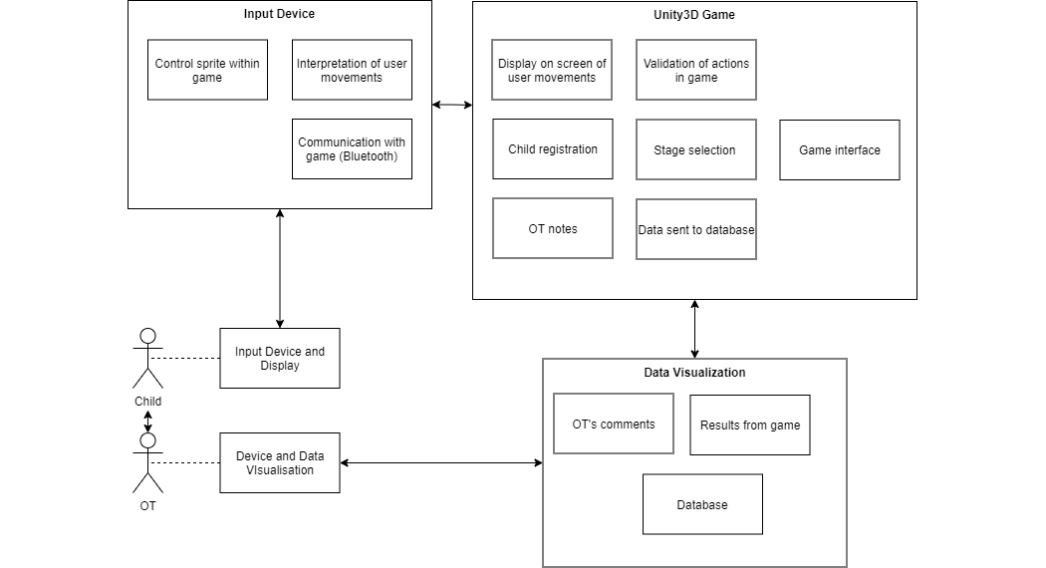
High-level design of solution. OT: occupational therapist.

**Figure 3:**
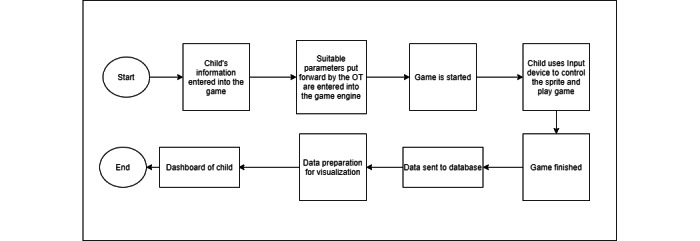
Sequence of events. OT: occupational therapist.

A high-level solution design is shown in Figure 2. The sequence of events is illustrated in Figure 3.

**Figure 4:**
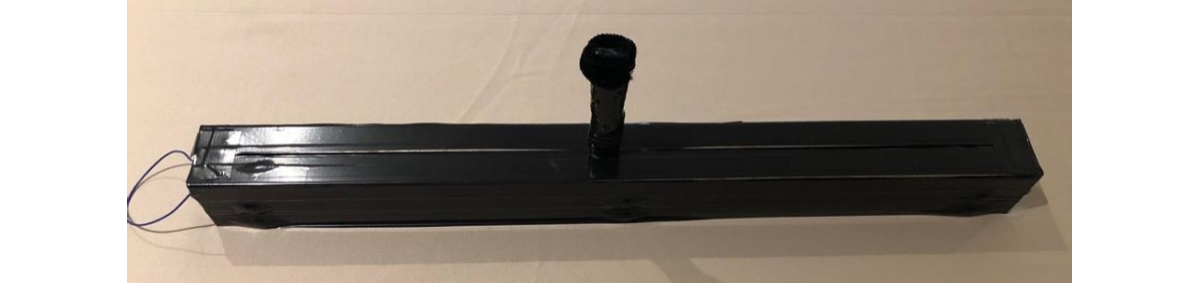
Prototype of input device.

The innovative low-cost input device shown in Figure 4 was developed previously and used so that the movement of the child could act as an input for the game [25]. The input device is designed in such a way that when the device is turned on and the game started, a connection is established. There are no complicated installations or setup steps. The role of the caretaker is to help the child set up the device with the game. Additionally, the caretaker will explain to the child how to play and then observe the child’s movements according to the guidelines designed by the occupational therapists, which will be discussed further in the *Results* section. The design of the data visualization component and the new serious game is presented in this study.

#### Initial Serious Game

The game genre chosen was a casual game because of its low system requirements, accessibility in most devices, and, at the same time, not demanding high levels of concentration from the player [26]. The game was not connected to the internet, and players had to install it to play it. The fact that the game does not take much concentration is an important factor in ensuring that the child does not become fatigued and frustrated. Moreover, the duration of the game was purposely designed to be short to retain the child's concentration. The developed game comprised a sprite controlled by the child [24]. Sprite is the terminology used to describe an avatar, shape, or character that the child has control over. The device was attached to the hand of the child, and the child moved the device in an arc shape with their arm extended in front of them while standing. The aim of the game was to collect faces falling at a calculated position from the sky. The movements in the game ensured that the child crossed the midline. The game intended to prompt the child to perform particular movements that occupational therapists would invoke when using traditional methods of treatment.

#### Input Devices

The problems encountered with the initial design of the input device and the detailed new design have been previously published [25]. For the sake of completeness, a short overview of the input devices is given in this section. In the initial input device, an accelerometer and magnetometer were used to obtain the movement of the child [25]. Tilting of the device was not the only issue that affected the feasibility of the design; before every game, a calibration was required to obtain the reference of the sensor. It did not seem to be the most suitable solution for therapists to expect the child to perform the calibration each time they wanted to play the game, ensuring that they do not tilt the sensor. Therefore, a more child-friendly design was needed that would be able to track the movement of the hand from side to side (crossing the body's midline) and transform the position into the game. In addition to accurately transforming the movement, a device that would not require calibrating the sensor was required. When discussions were held with an occupational therapist during the design phase, it was suggested that an input device that would be placed on a surface be designed. The idea behind this was to allow the child to move their hand from side to side and exhibit the same motion required when performing tasks that cross the midline, such as writing. Through extensive research into suitable sensors or electrical devices that could be used, a clean, innovative solution was reached. The design entailed the addition of a linear potentiometer; the input device is shown in Figure 4 [25].

### Objective Two: Evaluate Whether the Online Game Is Fit for Purpose

After the prototype was developed, it was crucial to evaluate whether this solution was a feasible option for occupational therapists and whether it was fit for purpose. This was achieved through semistructured interviews with occupational therapists. These occupational therapists all had between 5 to 10 years of experience. In addition, as many children have difficulty crossing the midline, these occupational therapists were competent to give feedback regarding the identification and treatment of midline crossing difficulties. Note that the occupational therapist who was part of the participatory design sessions was not included in the evaluation of the system.

The researcher took each of the participants through a presentation detailing the purpose of this research. The researcher conducted a demonstration of the full solution, taking the occupational therapist through the game and explaining how each stage of the game was played. The occupational therapists then had the opportunity to play the game and evaluate each stage on their own. A semistructured interview took place, allowing the researcher to further explore the insights provided by

the therapist. Each session was conducted individually to ensure unbiased and objective responses. The interview aimed to highlight the strengths and weaknesses of the solution as well as the suitability of administering the solution remotely. The interview provided the therapists with the opportunity to suggest changes and offer recommendations. The questions were purposefully left open-ended so as not to limit the therapist's responses. The main themes that needed to be established from the interviews can be seen in Textbox 2. The questions contained in each section can be seen in Textbox 3.

**Main themes established from interviews.**

**Main themes**

- The feasibility of using a serious game and input device to assist occupational therapists in identifying and treating children with midline crossing difficulties
- The benefits and applicability of the dashboard
- The applicability of the solution in areas where the availability of occupational therapy resources is limited

**Questions asked to occupational therapists during the interviews.**

**Questions**

- In your professional context, comment on whether a serious game and input device would assist you as a therapist to identify children with midline crossing difficulties as well assist in your treatment process. Please include in your answer the following:
  Advantages
  Disadvantages
  Suggested changes
  Recommendations
- Comment on whether the use of the dashboard is beneficial and applicable to assist you as a therapist in identifying whether a child has pathology with midline crossing. Does the dashboard provide constructive tracking of the child's progress during treatment?
  Advantages
  Disadvantages
  Suggested changes
  Recommendations
- Comment whether the solution can be reasonably implemented and used effectively in areas where occupational therapy resources are limited.
  Advantages
  Disadvantages
  Suggested changes
  Recommendations

## Results

### Objective One: Designing a Remote Monitoring System for a Midline Crossing Serious Game

#### Adapted Serious Game

The initial game, as described in the previous section, was used as a base for the new telehealth system. The new system comprised an adapted serious game (with distinct levels), an input device, and a web-based backend system that enabled occupational therapists to access dashboards and behavioral information about the intervention. During the collaborative design sessions, it was decided that the serious game would be divided into different stages. Each stage has a specific aim, and different game variables can be set according to each child’s individual needs. The game comprises 4 stages: an assessment stage, 2 intervention stages, and a maintenance stage [24]. In each stage, there are 3 variables: distance, speed, and time, which are used to create a specific environment for testing. The time variable, set at 2 minutes, was kept constant for all stages. The chosen duration was advised by the occupational therapist. The duration was purposely set to be short to allow the child to concentrate for a short amount of time without getting bored or losing focus. Each stage addresses a certain intervention, which will be explained below; therefore, the input to each stage is different. The occupational therapist can set these inputs however they see fit (or advice the caretaker at home on which values to use during remote treatment).

**Figure 5:**
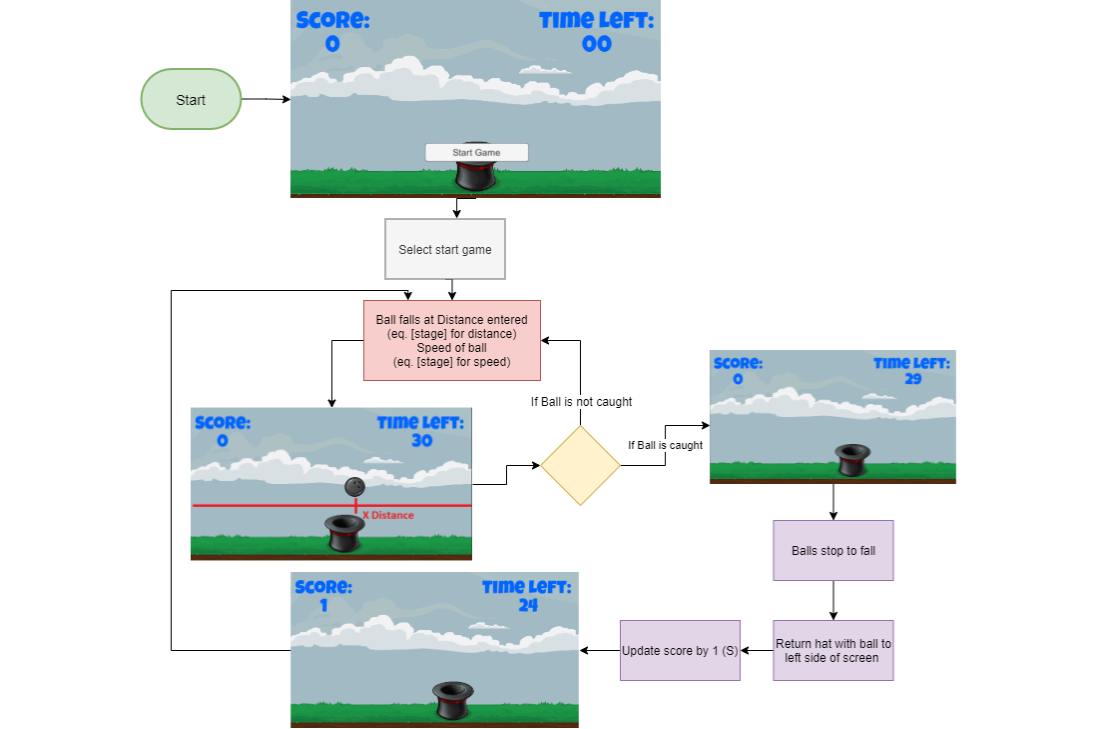
General game flow. eq: equation.

The game environment is chosen by selecting a stage and choosing the required values for the inputs for that stage. The game commences with the hat (controlled by the child using the input device) starting either on the left or right side of the screen depending on the handedness of the child. For the purpose of this explanation, a child who is right-handed will be used; therefore, the sprite starts on the left. If the child were left-handed, the sprite would start on the right so that the child’s first movement would be crossing the midline. The balls that fall from the sky are strategically placed, depending on the stage, which will be discussed below. When the balls fall, the child must catch the ball in the hat and return the hat to the starting point (either the left or right side of the screen) to earn a point. Textbox 4 shows the variables used in the equations. Table 1 shows the inputs and equations for the variable set. The game flow is illustrated in Figure 5; depending on the stage, the equations shown in Table 1 will be inserted.

**Variables used in the equations to set the game environment.**

**Variables**

- X_0_ is the starting distance
- I is the incremental distance
- S is the score
- DV is the distance variance
- Random (a,b) is the random function that provides a random number between a and b
- Sp_0_ is the initial speed
- Is_p_ is the incremental speed

**Table 1:** Equations used in each stage to set the game environment.

Stage	Input	Distance	Speed	Time
1	X_0_^a^, I^b^	X_0_+(IX_s_^c^)	Constant	Constant
2	X_0_, DV^d^	X_0_+Random (–DV, DV)	Constant	Constant
3	X_0_, Sp_0_^e^, Is_p_^f^	X_0_	Sp_0_+(Is_p_SX)	Constant
4	N/A^g^	Random (–7.5,7.5)	Constant	Constant

^a^X_0:_ starting distance.

^b^I: incremental distance.

^c^IX_S_: incremental distance as function of score.

^d^DV: distance variance.

^e^Sp_0:_ initial speed.

^f^Is_p_: incremental speed.

^g^N/A: not applicable.

#### Stage 1

**Figure 6:**
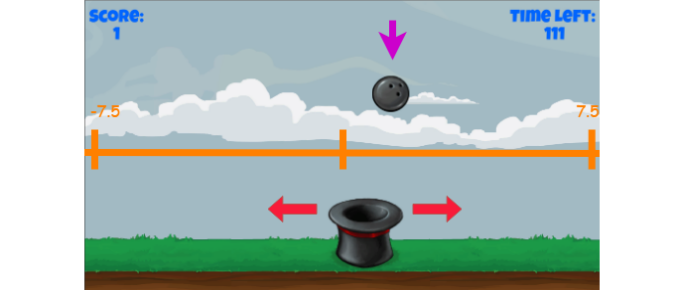
Position on x-axis in game.

Stage 1 takes the *starting distance* (X_0_) and *incremental distance* (I) as input. The starting distance is the point on the x-axis where the first ball will be spawned from the sky (top of the screen). The x-axis is depicted in Figure 6. The *incremental distance* is the distance that is incremented from the starting distance. The point on the x-axis where the balls are spawned is a function of the *score* (S); as the *score* increases, so does the distance of the spawned ball. The score represents the number of balls caught and returned. A ball is considered *returned* when the hat reaches the left side of the screen if the child is right-handed; if the child is left-handed, the hat would need to be *returned* to the right side of the screen. The score will update only after the hat has been returned. Consequently, the point at which the next ball falls will increase by the incremental distance (increase to the right if the child is right-handed). If a ball is missed, intuitively, the score will not increase, thus remaining the same; therefore, the ball will be spawned in the same place instead of being spawned at the incremented distance.

Stage 1, which is the assessment stage, aims to determine the child’s gross ability to cross the midline. In addition to the stages incorporated into the game, an interface to input the specified variables determined by the therapist was introduced. These variables create a tailored game environment for the child. In stage 1, the speed and time variables remain constant, whereas the distance variable is incremented. The input device shown in Figure 4 controls the movement of the hat illustrated in Figure 6 from side to side, simulating the movement of crossing the midline while writing. After the child catches the ball, the hat needs to be moved all the way to the other side of the screen, ensuring that the midline is crossed.

**Figure 7:**
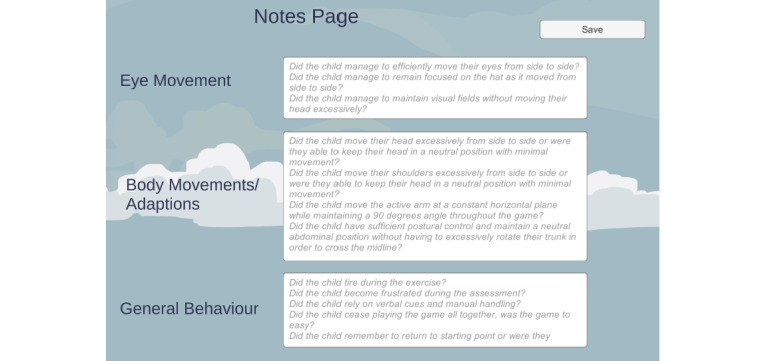
Notes page to fill in after each stage is completed.

A reference point for the child’s baseline functioning, including their ability to cross the midline, was established in stage 1. The amount of external input required to achieve midline crossing would be noted by the caretaker on the system. Although the game is designed to determine the core impairments of crossing the midline, clinical reasoning, observations, and external guidance from the occupational therapist are vital for a holistic and accurate assessment. In the event that an occupational therapist is not performing the assessment (ie, caretaker, parent, teacher, or guardian), a notes page, shown in Figure 7, will need to be completed, which is presented to the caretaker at the end of the game. A guidance script with key points of reference will be provided to the caretaker before the child plays the game to assist and ensure accurate observations. Using the results, an individualized treatment plan tailored to the child’s needs would be constructed and developed by the occupational therapist, which can be done remotely.

#### Stage 2

Stage 2 takes the *starting distance* (X_0_) and *distance variance* as inputs. Here, the *distance variance* is the distance from the *starting distance*, and the distance can be varied; for example, if the *starting distance* is 1.5 and the *distance variance* is 0.5, the ball can be spawned between points 1 and 2 on the x-axis.

Stage 2 is the first intervention stage, aimed at improving the child’s gross ability to cross the midline. This stage allows for the distance variable to be adjusted, whereas the speed and time variables are constant. The caretaker sets the distance at the specific distance where the child began experiencing problems with midline crossing. This distance can then be adapted and graded during the intervention.

#### Stage 3

Stage 3 takes the *starting distance* (X_0_), *initial speed* (Sp_0_), and *incremental speed* (Is_p_) as inputs. The *initial speed* is the speed at which the ball starts to fall. The *incremental speed* is the increase in speed from the starting point. The ball will continue to fall at the specified starting point; however, the speed at which the ball falls will increase as each ball is caught.

Stage 3, the second intervention stage, aims to enhance the child’s gross ability to cross the midline. The distance and time variables will remain constant, whereas the speed variable will be incremented. This increases sufficient accuracy and skill.

#### Stage 4

Stage 4 does not require any input as the position at which each ball is spawned is random. The point at which each ball is spawned is calculated using the random function *Rand()*. As shown in Figure 6, the x-axis spans from –7.5 to 7.5; therefore, the ball can be spawned at a random point on the axis. The speed of the ball remains constant and does not increase as a function of the score. The gameplay time is constant for stage 4.

The purpose of stage 4, the unregulated round, is to maintain the skills the child has obtained in the previous stages. In this stage, the speed and time variables will remain constant, whereas the distance variable will be random.

### Scoring

The occupational therapist advised on the scoring criteria for the stages during the collaborative design sessions. These scoring criteria were based on observations that occupational therapists would typically make during traditional treatments. The first three criteria were generated from the gameplay data received from the game, and the following five criteria were recorded by observation. To confirm that the child did not display any form of pathology with midline crossing, a child would need to achieve a total of ≥4 points on their score card. It should be noted that all 8 criteria should be used in conjunction with one another and not assessed independently. Table 2 shows the 8 criteria, the observation being assessed, and the measurement to score the criterion for stage 1. If a child meets the scoring for a criterion, a point will be awarded. The criteria for the other stages are the same as those for stage 1, except for criterion 4. In the scoring criteria for stage 2, the effect of the random displacement of balls in a targeted area is determined rather than the increasing distance. In the scoring criteria for stage 3, the effect of increasing the speed of the balls is determined rather than increasing the distance. Finally, in the scoring criteria for stage 4, the effect of the balls randomly falling is determined.

**Table 2:** This table shows the scoring criteria for an assessment (stage 1).

Criterion	Observation	Scoring
1	Balls caught	>14
2	Balls missed	<4
3	Average time between balls	<7.5
4	Distance increasing	No pattern
5	Follow instructions	Yes
6	Eye movements	Maintain visual focus
7	Body movement	No gross adjustments
8	General behavior	No verbal cues

### Data Visualization

**Figure 8:**
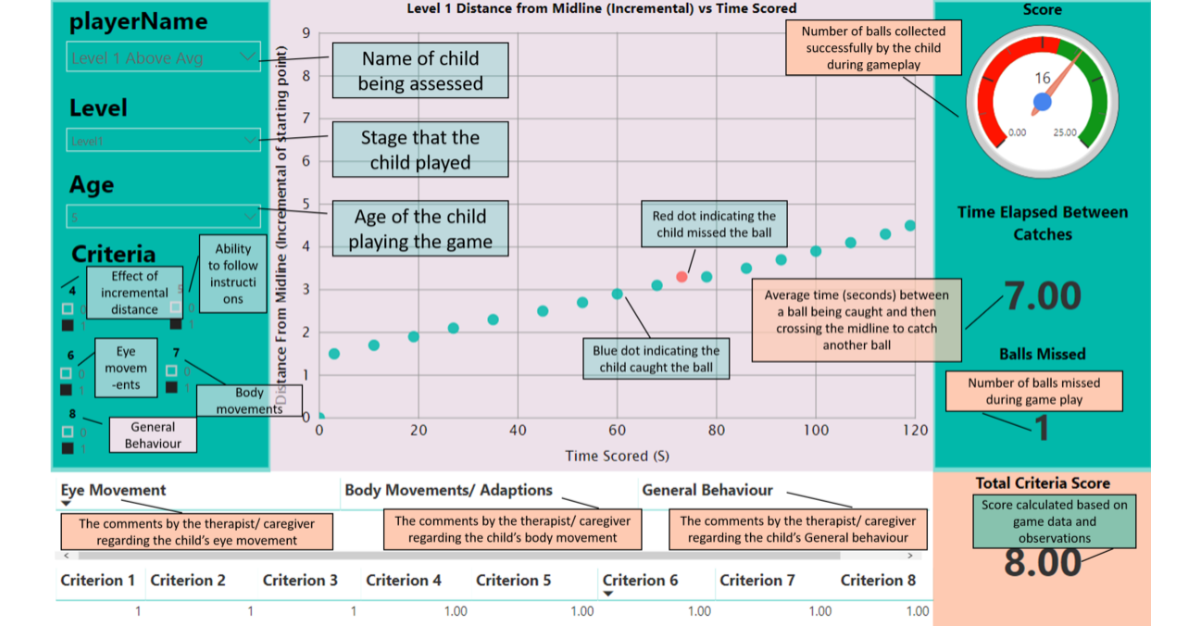
Representative dashboard showing how the tooltips will be displayed using Microsoft PowerBI.

Occupational therapists can support children with difficulty crossing the midline by helping them develop the skills needed to perform activities of daily living. During therapy sessions, occupational therapists use a variety of techniques to support rehabilitation, such as functional electrical stimulation, constraint-induced motor training, facilitation, and virtual reality applications [27]. This combination of therapy, exercise, and context-specific retraining is critical for neuroplasticity, as mentioned in the *Introduction*. Although retrospective recall and exercise diaries can gather subjective data, the quality of these data is limited and relies on the notes and observations written by an occupational therapist; as such, occupational therapists lack objective data about the degree to which exercises have been performed. Consequently, a dashboard was designed to aid occupational therapists by providing a collection of objective data about children who have difficulty crossing the midline. The proposed monitoring tool provides a solution in which the gameplay of the child during an assessment or treatment is recorded, and the data collected in the game can be visualized by the therapist. The design of the system, and the monitoring functionality in particular, allows for the game and input device to be used by parents or guardians with the children in a home environment and to be used remotely. Therefore, the game would not be restricted to only being played during therapy sessions but rather can be played outside of these times as well. A dashboard was designed using Microsoft’s PowerBI to display the data illustrated in Figure 8.

**Figure 9:**
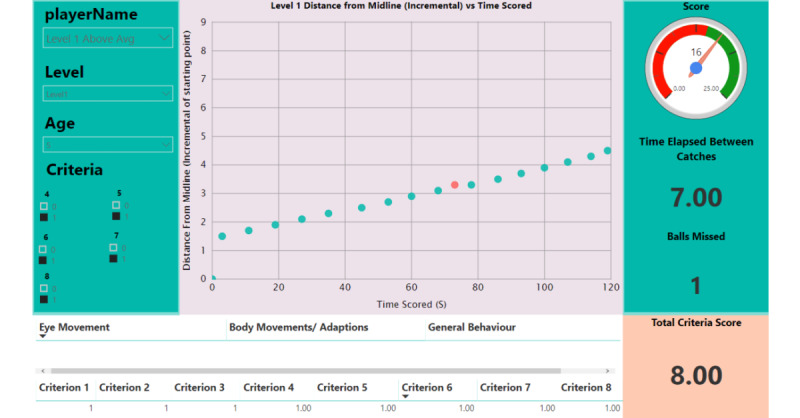
Representative dashboard for a child whose performance was above average for stage 1.

**Figure 10:**
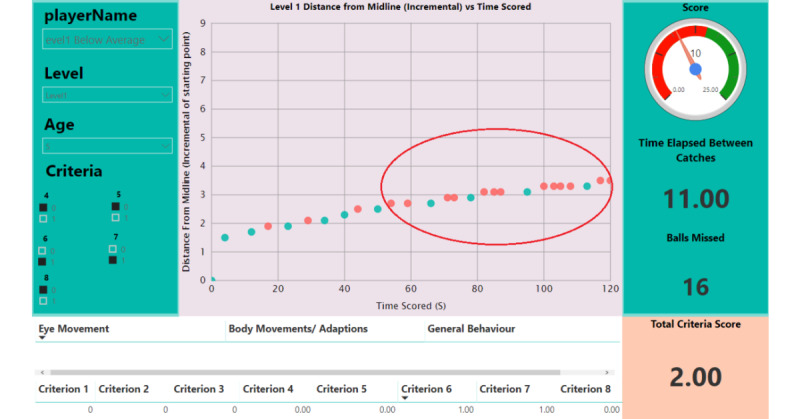
Representative dashboard for a child whose performance was below average for stage 1.

During the evaluation process, the occupational therapists were asked to play the game themselves and get familiar with how the system works. Figure 9 shows an illustrative example of how the dashboard would look for a child without midline crossing difficulties. The dashboard is accessible remotely. Figure 10 is an illustrative example of a dashboard for a child with midline crossing difficulties. The red circle indicates that as the distance increased, the *child* missed more balls. In this case, there are various factors that could impact the *child’s* results. For example, the *child* may have felt bored and, therefore, did not play as they should have. A child with an average performance result needs to be further assessed to see whether their results are because of pathology with midline crossing or whether there are other factors yielding these results.

### Objective Two: Evaluate Whether the Web-Based Game Is Fit for Purpose

The therapists commented on the convenience of integrating both assessment and treatment into the same application as it assists the therapists when grading a child. The stages developed in the game were also commended by the therapists, as each stage has a different focus point with a specific outcome. They collectively agreed that the quantitative aspect that the game creates by providing measurable and standardized data proves advantageous when compared with traditional methods of assessment and treatment. When using a traditional method, such as asking the child to build a puzzle or draw a line across a page, there are no quantitative measures that can be deduced; instead, the assessments are limited to mere observation. The proposed solution elicits not only quantitative data but also allows observational data to be recorded using the notes page.

Furthermore, they referred to the notes page as not only descriptive but also appreciated that it was designed to be understood by nonmedical professionals, thereby making the observational recording more effective and user-friendly. The notes page is viewed as an extremely beneficial aspect of the serious game as it ensures that the assessor covers all the observational components included in the assessment and treatment. As a result of the movements triggered by the game and the quantitative and observational data recorded by the application, the occupational therapists concluded that the serious game, accompanied by the input device, would assist them in identifying whether a child has pathology with midline crossing. Moreover, the solution would also assist in treating the child’s pathology. Finally, the therapists thought that the solution could assist in exposing and identifying other gross and fine motor pathologies that a child might have, or it might even reveal underlying behavioral, cognitive, or physical impairments. It was suggested that the assessment stage should be standardized per age group.

One of the ways to identify whether a child has pathology with midline crossing is by observing whether the child has a midline jerk. This can be noticed when the child is performing actions where the midline is crossed, and their eyes move from side to side in a rapid manner. Consequently, the therapist proposed incorporating eye tracking to detect if the child’s oculomotor functions are impaired. Additional monitoring through the use of a laptop camera can also be provided to observe the eye movement, head adjustments, and posture of the child.

As part of the game, when the child catches the object falling in the hat, the child is required to return the hat to the side of the screen to receive a point. Even though an alert is sounded when the object returns to the side, a suggestion was proposed to display a reminder on the screen to return the object to the side at the point where the child has caught the object. In addition, when the child is playing the game and controlling the input device, the child may release the device. Consequently, it was recommended that if the child releases the device during the game, an alert should be shown to remind the child to grasp the device.

Specific attention was given to the dashboard design as it is an important part of enabling remote monitoring. Everyone unanimously agreed that the dashboard’s ability to track the progress of the child would benefit their reports immensely. The results represented in the dashboard would assist them in supporting and verifying their observational conclusions. Furthermore, when submitting reports to their clients’ medical aids, the data from the dashboard can be used to solidify their conclusions and can be included in their reports.

The only disadvantage that was raised by the occupational therapists was that the dashboard might not be particularly easy to interpret for a layman; however, a suggestion was made to provide training to therapists on how to interpret and analyze the dashboard. The other disadvantage was that the dashboard was limited to only showing the results for a midline crossing assessment and no other difficulties that could be identified by the game.

There were suggestions to provide a way of showing the standardized results for different categories by searching by age, for example, and, consequently, the results for that particular age group would be displayed. Introducing standardized norms would allow the child to be compared with other children within the specific area being examined. Another suggestion that was made was to allow therapists to add additional comments on the results of the child displayed for further reference. Finally, it was proposed that artificial intelligence could be incorporated so that when comments are made on the notes page by the therapist or guardian, the observations can be translated into quantitative data that can be used to calculate the scoring criteria instead of physically inputting the scores for the criteria that require observations.

The occupational therapists agreed that this solution could be used as a telehealth device. To further enhance these benefits, it was suggested that the game be deployed as a mobile app. This would allow for the game to be available on smart devices, which would, therefore, make it even more accessible than if it were only available on a laptop or computer. A second proposition was to record the gameplay so that it could be played back for reference. It was recommended that a video or chat capability be introduced so that the child or guardian could communicate with the therapist.

## Discussion

### Principal Findings

A telehealth system consisting of a serious game accompanied by an input device and a dashboard can be implemented to address children’s midline crossing difficulties. The scoring system provides a quantitative aspect that proves advantageous when compared with traditional methods of assessment and treatment, where the assessment is limited to mere observation. In addition, the notes page that is completed at the end of the serious game ensures that the observational components that are vital in assessing a child are still included in the overall assessment. Therefore, the telehealth system elicits not only quantitative data but also allows observational data to be recorded using the notes page.

Although there are many input devices that can be used for therapeutic reasons, as described in the literature section, they each have shortcomings that would need to be addressed to make them more suitable when used by a child with a pathology with midline crossing. The common drawback of all the devices is their cost. It is not feasible to expect parents to buy expensive devices for treatment at home that will only be used for a limited time. A Kinect system will cost approximately US $399 and a Wii, US $164, whereas the proposed system costs approximately US $32 to manufacture. This price may also decrease if large quantities are manufactured.

The Wii provides a suitable solution for physical training and balance in particular; however, there are no games that explicitly aim to treat or assess children with midline crossing [18]. At first, Leap Motion seemed to be a suitable device for the development of a VT; however, with inaccurate readings stemming from the device affecting the game experience, the device would not be suitable for children [21]. The RealSense is a fitting tool; however, because of the high price of the device, it is not suitable [19].

Studies with a serious game and Kinect showed that individuals are more motivated, enjoy therapy, and even enable therapy to be more accessible [12]. On the basis of the feedback from the occupational therapists, the child will also experience these benefits from the system presented in this paper. However, this will only be proven in clinical studies.

### Limitations

When determining whether serious games are feasible as a treatment option for midline crossing difficulties and are fit for purpose, there appears to be a lack of clinical evidence about the benefit to children from the application of serious games [28]. Owing to ethical constraints regarding testing the solution directly on children, professional opinions of occupational therapists were gathered to validate the solution. The next step would be to obtain the needed clearance to test directly on children without difficulties crossing the midline to attain a baseline. Thereafter, tests can be performed on children who have difficulty crossing the midline. Although the dashboard was configurable, it was found to be slightly difficult to interpret. It was suggested that therapists could be trained to interpret and analyze the dashboard. The telehealth system focuses on only one intervention. Therefore, the possibility of using the serious game and input device to assist in exposing and identifying other gross and fine motor difficulties that a child might have could be investigated.

### Future Work

The therapists were confident that the telehealth system presented will assist them in identifying and treating children with midline crossing difficulties. Therefore, the possibility of using this solution to expose and identify other gross and fine motor difficulties that a child might have could be investigated. Furthermore, the solution could even reveal underlying behavioral, cognitive, or physical impairments. To clarify, the therapists would be able to identify other aspects, such as hand dominance, eye tracking, postural control, range of motion, attention and focus of the child, and the child's hand functions (grips and grasps) when playing the game. Furthermore, when identifying whether a child has midline crossing difficulties, a midline jerk can be observed. When a child is confronted with actions in which the midline is crossed, their eyes move from side to side rapidly. As a result, adding eye-tracking capabilities to the solution was put forward. To mitigate the concern raised by the therapists regarding the fact that an older child may achieve the game objectives quicker than a younger child, a standardized assessment stage per age group can be introduced.

One of the actions required in the game when a falling object is caught is to return the object to the side to receive a point. Currently, when the object is returned, an alert is sounded, indicating that a point is scored. A suggestion was proposed to display a reminder to return the object to the side. Moreover, when the child is playing the game using the input device, the child may release the device. Consequently, it was suggested that in the event that the device is released during the game, an alert should be displayed to remind the child to remain holding the device. In addition, the results presented on the dashboard are limited to displaying the results for 1 player at a time. A recommendation was proposed to provide a way of showing standardized results for different categories, such as age. This would allow the child to be compared with other children within a specific area examined.

In future iterations, artificial intelligence can be incorporated so that when comments are made on the notes page by the therapist or guardian, the observations can be translated into quantitative data that can be used to calculate the scoring criteria instead of physically inputting the scores for the criteria that require observations. Finally, the solution proposed in this dissertation extracts gameplay data that can assist occupational therapists in identifying and treating children with difficulty crossing the midline. A greater number of occupational therapists adopting the solution in their practices means that more children will play the game and, ultimately, means more data are accumulated. These data can be analyzed using machine learning algorithms to find trends and enhance the assessment and treatment processes and visualizations produced.

### Conclusions

A solution was designed to determine whether a telehealth system comprising a serious game can assist occupational therapists in identifying whether a child has pathology with midline crossing and can assist in treating the child remotely. Serious games are introduced as a need to meet objectives that go beyond entertainment and benefit the user in the area that needs to be mitigated. Through collaboration with occupational therapists, the telehealth system was designed to make use of different levels in the serious game, where each level addressed a different need of the therapy process. A novel, low-cost input device accompanies the serious game to track the movement of a child’s hand from side to side and transform the position into the game. All results are saved on the web, and occupational therapists can access a dashboard that displays the results of each child. In addition, observed behavioral information will also be saved to assist occupational therapists in making decisions regarding changes to the intervention. During the interviews, occupational therapists indicated that the dashboard would support their treatment plan and that the end-to-end solution was indeed feasible.

## Abbreviations

IMU: inertial measurement unit
VT: virtual therapist
